# COVID-19 Conspiracy Theories Discussion on Twitter

**DOI:** 10.1177/20563051221126051

**Published:** 2022-10-10

**Authors:** Dmitry Erokhin, Abraham Yosipof, Nadejda Komendantova

**Affiliations:** 1 International Institute for Applied Systems Analysis, Austria; 2 Faculty of Information Systems and Computer Science, College of Law & Business, Ramat-Gan, Israel

**Keywords:** COVID-19, conspiracy theory, infodemic, Twitter

## Abstract

The coronavirus disease 2019 (COVID-19) pandemic was an unexpected event and resulted in catastrophic consequences with long-lasting behavioral effects. People began to seek explanations for different aspects of COVID-19 and resorted to conspiracy narratives. The objective of this article is to analyze the changes on the discussion of different COVID-19 conspiracy theories throughout the pandemic on Twitter. We have collected a data set of 1.269 million tweets associated with the discussion on conspiracy theories between January 2020 and November 2021. The data set includes tweets related to eight conspiracy theories: the 5G, Big Pharma, Bill Gates, biological weapon, exaggeration, FilmYourHospital, genetically modified organism (GMO), and the vaccines conspiracy. The analysis highlights several behaviors in the discussion of conspiracy theories and allows categorizing them into four groups. The first group are conspiracy theories that peaked at the beginning of the pandemic and sharply declined afterwards, including the 5G and FilmYourHospital conspiracies. The second group associated with the Big Pharma and vaccination-related conspiracy whose role increased as the pandemic progressed. The third are conspiracies that remained persistent throughout the pandemic such as exaggeration and Bill Gates conspiracies. The fourth are those that had multiple peaks at different times of the pandemic including the GMO and biological weapon conspiracies. In addition, the number of COVID-19 new cases was found to be a significant predictor for the next week tweet frequency for most of the conspiracies.

## Introduction

The coronavirus disease 2019 (COVID-19) pandemic was a new kind of risk when emergency lasted for a longer period. It caused disruptions in many aspects of human life and lead to long-term psychological consequences (Fahriani et al., 2021). Decision makers had to act in conditions of uncertainty. Information and data about the virus and its spread were missing due to the new character of the risk which impacted several countries. In addition, in comparison to other crisis situations, social media were playing a decisive role during the COVID-19 pandemic. People were searching and exchanging information, also in condition of the lack of evidence or scientifically proofed data, especially during the first phases of the pandemic. This situation resulted in a real infodemic. The term appeared in 2002 to describe the science of studying “the determinants and distribution of health information and misinformation” (Eysenbach, 2002). In 2003, the term was used in connection with the severe acute respiratory syndrome (SARS) epidemic for a situation where “a few facts, mixed with fear, speculation and rumor, amplified and relayed swiftly worldwide by modern information technologies” affected the economy, politics, security, and society (Rothkopf, 2003). With the onset of the COVID-19 pandemic, the term infodemic was reintroduced by the United Nations (UN, 2020) and World Health Organization (WHO). The infodemic had a direct impact on risk perceptions of the COVID-19 pandemic and on risk mitigation actions, such as vaccines, and resulted also in various kinds of behavior in emergency. Various so-called conspiracy theories were spreading, mainly about the source of the COVID-19 virus, the effectiveness of vaccines, and their side effects. Social media became one of the main sources of their spread.

Although there is a, albeit limited, literature on the topic of COVID-19 conspiracies, most prior work focuses on single conspiracy theories and is limited in time and volume of data analyzed. Against this background, this article contributes to the analysis of the most common conspiracy theories over the course of the pandemic from January 2020 to November 2021. Our aim is to understand the evolvement of the discussion of various conspiracy theories related to the COVID-19. Considering the large volumes of information, we have selected eight conspiracy theories: the emergence of COVID-19, which involves theories such as fifth-generation technology standard for broadband cellular networks (5G), genetically modified organism (GMO), the role of Bill Gates, the Big Pharma industry, and biological weapons; the scope of the pandemic, which involves theories such as empty hospitals and exaggeration; and theories about the effectiveness and side effects of vaccines. For each of these conspiracy theories we have identified a set of keywords and used the Twitter’s Application Programming Interface (API) to extract about 1.2 million English-language tweets from early January 2020 until November 2021. More specifically, we have analyzed the temporal distribution of tweets by different theories. This has allowed us to instantly determine when the discussion of which theory prevailed and how the distribution has changed over time. Given the relatively long period of time since the beginning of the pandemic, the continuation of the discussion of conspiracy theories may indicate a lack of information work to dispel them. COVID-19 is a type of severe crisis where, according to the situational crisis communication theory, providing objective information only is not enough, but proper crisis response strategies are required (Paek & Hove, 2019).

The article is structured as follows. The second section introduces literature on the COVID-19 conspiracies and provides a theoretical framework for conspiracy theory beliefs. The third section examines the conspiracy theories explored in the article and describes collected data and methodology. The fourth section presents the results, which are discussed in the fifth section. Finally, the sixth section concludes the article.

## Literature Review

### COVID-19 Conspiracies

Social media, particularly Twitter, have played a prominent role in the dissemination of conspiracy theories. This has given rise to a number of papers analyzing the spread of conspiracy theories. That said, studies diverge in their findings about the role of conspiracy theories in the COVID-19-related tweets. The share of conspiracy tweets ranges from 0.6% to 18%. Shahrezaye et al. (2020) analyze the spread of conspiracy theories in the German-language segment of Twitter. Shahrezaye et al. (2020) found that less than 1% of 9.5 million COVID-19-related tweets are on the conspiracies. Out of about 4,900 tweets, Nuzhath et al. (2020) identify about 18% as conspiracy theories. Li et al. (2020) determine that 2% of 7000 tweets are conspiracies. Moffitt et al. (2021) recognize about 1.5 million tweets out of about 244 million as conspiracies. Jamison et al. (2020) find that 9% of 1,689 accounts write about conspiracies.

We contribute to this strand of literature by going one step further and looking into the conspiracy tweets. We are looking at the narrative of the full discussion about the conspiracy theory with the supporters, the opponents, and the neutral side. Our interest is not in measuring the number of conspiracy tweets relative to the total number of tweets, but in analyzing the discussion on conspiracy tweets themselves: what specific theories are discussed, to what extent, and how the discussion frequency changes over time.

A number of papers explore the spread of single conspiracy theories. Ahmed, Vidal-Alaball, et al. (2020) study the 5G conspiracy. They analyze 233 tweets with a 5G hashtag and find that about one third come from the supporters of the conspiracy theory. Gruzd and Mai (2020) look at the FilmYourHospital conspiracy and analyze about 100,000 related tweets. What makes their study special is that they show how this conspiracy theory originated from just one tweet. Ahmed, Seguí, et al. (2020) also study the FilmYourHospital conspiracy with the goal to determine drivers behind it. After analyzing about 23,000 related tweets, they found that ordinary citizens are the most important drivers of this conspiracy. Kearney et al. (2020) focus on the plandemic (planned pandemic). They analyze about 85,000 tweets and reveal how the appearance of a conspiracy documentary about a planned pandemic affected the discourse. Visentin et al. (2021) look at 5,615 tweets related to the Italian tracing app “Immuni” and study the conspiracy on this app being a plan to limit people’s freedom. They classify 21% of the tweets as related to the conspiracy.

We contribute to this strand of literature by analyzing the discussion on eight different conspiracy theories. This makes it possible not only to estimate the number of conspiracy related tweets, but also to compare the theories among themselves and identify patterns in their discussion.

Another group of studies touches upon various conspiracy-related topics, which are worth mentioning briefly. Ferrara (2020) looks at the role of automated accounts—bots in spreading conspiracy theories. An analysis of more than 43 million English COVID-19-related tweets reveals that bots are used to promote political conspiracies, whereas human accounts are more concerned with public health issues. Moffitt et al. (2021) find that news sites with low fact-checking scores support conspiracies, and their spread is strengthened by bots linked to prominent Twitter users. Stephens (2020) examines the geospatial distribution of conspiracies. Gerts et al. (2021) estimate the sentiment of conspiracy tweets. They detect a more negative sentiment among misinformation tweets and show the evolvement of theories over time with the inclusion of details from other unrelated conspiracies and real events. Papakyriakopoulos et al. (2020) discuss the content moderation of various conspiracies by social media platforms.

### Risk Perceptions

Risk perceptions play a large role in the emergence and proliferation of conspiracy theories related to COVID-19. Conspiracy theories emerge as an attempt to describe hard to explain and unexpected events, into which group COVID-19 fits very well. It represents a typical example of the so-called dread risk—low-probability and high-consequence catastrophic event difficult to control (Deerberg-Wittram & Knothe, 2020; Leitner, 2021; Slovic, 1987). It struck unexpectedly, has already resulted in a large number of deaths and illnesses, can hardly be controlled, and has lasting behavioral consequences. Such risks tend to be overestimated by ordinary people, who fear them more than some highly probable and dangerous events like car accidents. There is an immediate human response to such risks, which is consistent with the fast thinking and can lead to an inability to distinguish between facts and fiction (Kahneman, 2011; Xu et al., 2020).

The massive and rapid use of social media during the pandemic has become both an aid and an additional threat (Venegas-Vera et al., 2020). Dissemination of misinformation reinforces the perception of COVID-19 as a dread risk and can provoke inappropriate human behavior, which leads to even greater spread of the virus. We can draw a parallel with the terrorist attack of 11 September 2001, which is also an example of a dread risk. In response, people became afraid to fly, tried to minimize the use of airplanes and increased the use of automobiles. However, research shows that the number of people who switched from planes to cars and died on the road is greater than the number who died in the fatal 9/11 planes (Gigerenzer, 2004). The same could be seen with COVID-19 when, for example, rumors of lockdowns in the northern Italy caused people to want to leave to the southern Italy, resulting in crowds in railway stations and airports and a markedly increased risk of infection and contamination (Cinelli et al., 2020).

West (2015) discusses some of the dread risk biases related to a nuclear accident, which can also be used to describe the expectations associated with COVID-19. According to the heuristic-systematic model, individuals act heuristically in response to threats, that is, based on their emotions, and according to the group epistemological theory, these individuals also group together around similar labels. This is perfectly visible in the emergence of COVID-19 conspiracy theories. At the same time, individuals react heuristically, trying to find a seemingly logical explanation for the emergence of the pandemic, as is discussed below, and band together on the principles of attitudes toward different aspects of the pandemic, such as the vaxxer group and the anti-vaxxer group. The threat and distrust heuristic is invoked to deal with industrial power and related fears about the role of big industry. We see a similar thing with COVID-19, where proponents of conspiracy theories attribute a large role to the so-called Big Pharma. Furthermore, using the argument of pure progress to create trust can have the opposite effect and turn away half of the audience, as we also observe today when medical progress is perceived, among other things, as attempts at chipping and universal control. A further important role is played by the most popular perceptions (e.g., in the nuclear industry, that reactors could potentially fall victim to a runaway reaction), which results in scientists having to reckon with these public narratives, regardless of how scientifically sound they may be. It is the same with COVID-19, where the scientific community is forced to respond to the most unexpected public narratives, such as the link between COVID-19 and 5G.

## Methods and Data

### Conspiracy Theories

To analyze the evolvement of the discussion of conspiracy theories during the pandemic, we first need to define them. Under a conspiracy theory, we understand “a subset of false narratives in which the ultimate cause of an event is believed to be due to a malevolent plot by multiple actors working together” (Swami, 2012). Conspiracy theories promote resistance to vaccines and preventive measures and create barriers to gaining public support for measures to prevent the spread of infection (Romer & Jamieson, 2020). Some of the most prominent include the conspiracies on the origin of the virus as well as on vaccine effectiveness and side effects. This being said, it is worth noting that there is no clear distinction between some conspiracy theories, and they may overlap.

Considering the large volumes of information, we have selected eight conspiracy theories: the emergence of COVID-19, which involves theories such as 5G, GMO, the role of Bill Gates, the Big Pharma industry, and biological weapon; the scope of the pandemic, which involves theories such as empty hospitals and exaggeration; and theories about the effectiveness and side effects of vaccines. Below, we briefly summarize conspiracy theories of interest for our research.

#### The 5G Conspiracy

The launch of 5G networks coincided with the outbreak of the COVID-19 pandemic (Ahmed, Vidal-Alaball, et al., 2020). Social media users began actively spreading the word that the two events were connected, even leading to residents in a number of British cities physically damaging 5G network towers to stop the spread of the virus.

#### The Big Pharma Conspiracy

Conspiracy theorists believe that big pharmaceutical companies are behind the spread of COVID-19, and that people like Bill Gates or Dr Fauci are acting on their behalf (Ali, 2020; Jamieson, 2021).

#### The Bill Gates Conspiracy

A non-negligible number of people around the world believe that Bill Gates played a role in the creation and distribution of COVID-19 for the purpose of microchipping people (Thomas & Zhang, 2020). Some of the possible reasons for the theory discussed in the literature are 2019 events connected with Gates associated companies such as pandemic simulations, the registration of a patent with three sixes in the title on a cryptocurrency system that uses body activity data, the launch of a digital identity program, or also a 2015 Technology, Entertainment, Design (TED)-talk, where Bill Gates was warning of a viral outbreak (Ali, 2020).

#### The Biological Weapon Conspiracy

There are widespread conspiracy theories that view COVID-19 as a biological weapon, whether of Chinese (Nie, 2020), Jewish (Gerstenfeld, 2020), US (Jia & Luo, 2021), or any other origin.

#### The Exaggeration Conspiracy

Some conspiracy theorists believe that the scope of the pandemic is exaggerated, and some even believe that the coronavirus does not exist (Allington et al., 2020).

#### The FilmYourHospital Conspiracy

Especially at the beginning of the pandemic, there was a widespread theory that hospitals were actually empty, hence the scope of the pandemic was markedly exaggerated (Ahmed, Seguí, et al., 2020). People were encouraged to go to local hospitals and take pictures to show that they were empty.

#### The GMO Conspiracy

According to this conspiracy, genetically modified crops led to the emergence of COVID-19 (K. Chen et al., 2020).

#### The Vaccines-Related Conspiracies

A number of conspiracy theories are associated with vaccines: vaccines make people infertile, vaccines do not work, vaccines cause autism, vaccines lead to autoimmune disease, and others (Ullah et al., 2021).

### Data Collection

To examine the development of the conspiracy theories described above, we have collected data using Twitter’s API’s. We have used the Twitter API’s v2 full search endpoint which is limited to Academic Research. According to the API documentation, a full search query retrieves tweets matching the specified criteria throughout Twitter’s history up to March 2006 when the first tweet was published on the platform. The search criteria have included all tweets that contain one or more COVID-19 related keywords, and conspiracy-specific keywords, according to research articles (E. Chen et al., 2020; K. Chen et al., 2020; Jiang et al., 2020; Memon & Carley, 2020; Shahsavari et al., 2020). The full search query we have used is: “covid” OR “coronavirus” OR “corona” AND conspiracy-specific keyword, see Table 1 for the specific conspiracy keywords. The keywords maintain that we only collect tweets that are in some way related to the topic. Retweets have been excluded from the search. We have further limited the searches to tweets that have been determined by Twitter’s language detection algorithm to have been written in English. The criteria aim to provide all tweets within the scope of the conspiracy theories that are either supporting, opposing, or neutral of the conspiracy theory. We have collected all tweets published between 1 January 2020, shortly after the outbreak was first reported in the media, and 30 November 2021.

**Table 1.** Conspiracy Theory Keywords Used in This Study to Identify Tweets Discussing the Conspiracy Theory.

Conspiracy theory	Keywords
5G	5G
Big Pharma	(big pharma) or (fauci pharma) or (gates pharma)
Bill Gates	Bill Gates
Biological weapon	weapon
Exaggeration	(does not exist) or (doesn't exist) or (exaggerated) or (inflated)
FilmYourHospital	(FilmYourHospital) or (film your hospital) or (empty hospital) or (empty bed)
GMO	(GMO) or (genetically modified)
Vaccines	vaccine and {(infertile) or (do not work) or (don't work) or (does not work) or (doesn't work) or (autism) or (autoimmune)}

GMO: genetically modified organism.

The full data set contains about 1.2 million tweets, all of which match the criteria defined for at least one of the eight conspiracies (see Table 2).

**Table 2.** Total Number of English Tweets per Conspiracy Between January 2020 and November 2021.

Conspiracy	Number of tweets
5G	326,035
Exaggeration	314,205
Weapon	226,882
Big Pharma	173,452
Bill Gates	138,061
Vaccines	65,472
GMO	18,090
FilmYourHospital	7,054
Total	1,269,251

GMO: genetically modified organism.

To analyze how the discussion of various conspiracies evolved with the number of new cases we use the global number of new COVID-19 cases on a daily basis compiled by the Johns Hopkins University Center for Systems Science and Engineering (Dong et al., 2020). The university collects data from a large number of sources around the world, such as country health ministries. The data represent the number of new cases per day worldwide. For example, according to the data, on 8 July 2021, there were 480,205 new cases worldwide. The data allow covering the period between 23 January 2020 and 30 November 2021.

### Statistical Methods

To evaluate the discussion on each conspiracy, we present each one of the conspiracies as a time series of the tweet frequency over time. We conduct correlation and cross-correlation analysis between the conspiracy tweet frequency time series. This helps to identify dependencies between different conspiracies discussion.

We also apply the ordinary least squares (OLS) time series model to estimate the effect of new daily cases on the discussion of each of the conspiracies according to equation (1)

(1) $$\ln(y_{i,t})=\alpha_{i}+\beta_{i}ln(x_{t-7})+\varepsilon_{i,t},$$

where $y_{i,t}$ is the number of tweets related to the conspiracy *i* on day *t*, and $x_{t-7}$ is the number of new cases on day *t*−7, that is, a week before. We run the regression on a weekly basis because we expect a lag between the number of new cases and behavioral response, which can also be explained by the way the data on new cases are published with a delay. We log the data to make our model invariant to the scale of the variables, to have a much less heteroscedastic or skewed distribution of the variables, and to limit the effect of outliers.

To take into account the heteroscedasticity of the residuals, we use robust standard errors. Given that we have only one independent variable in each regression, the independence assumption is automatically fulfilled. Rationally, we would expect that the discussion of the conspiracies should decline with the number of new cases given that more people come into contact with the virus.

## Results

Table 2 presents the number of tweets related to each of the eight conspiracies collected between January 2020 and November 2021. We see that the 5G and the exaggeration conspiracies were the most discussed with more than 300,000 tweets followed by the weapon conspiracy with about 230,000 and the Big Pharma conspiracy with slightly more than 170,000 tweets. Then come the Bill Gates conspiracy with around 140,000 tweets and the vaccines-related conspiracies with 65,000 tweets. The GMO conspiracy with 18,000 tweets and the FilmYourHospital conspiracy with 7,000 tweets were the less discussed ones.

### The Conspiracy Theories Tweet Frequency During the Pandemic (January 2020 to November 2021)

**Figure 1.**
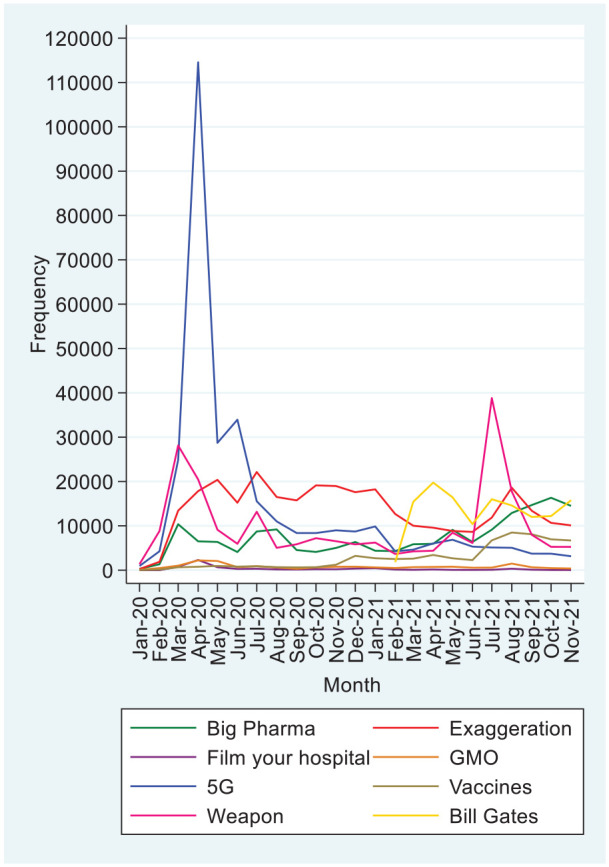
The monthly tweet frequency for each conspiracy theory.

Figure 1 presents the evolvement of the discussion of the conspiracy theories over time from January 2020 through November 2021 by month. The graphical analysis helps categorize conspiracy theories into four groups, which are then examined econometrically.

#### Peak at the Beginning of the Pandemic

The first group includes the 5G and the FilmYourHospital conspiracy theories. We see a peak in April 2020, a sharp decline straight afterwards and then a gradual decline of the theory (see Figure 1). The FilmYourHospital conspiracy theory follows a similar pattern with a peak in April 2020 and quite a sharp decline afterwards. From Table 3, we can see a positive and significant correlation (*r* = .134, *p* < .01) between the two conspiracies.

**Table 3.** Correlation Matrix Between the Conspiracy Theories Tweets Frequency Time Series.

	5G	Bill Gates	Big Pharma	Weapon	Exaggeration	FilmYourHospital	GMO	Vaccines
5G	1							
Bill Gates	0.293***	1						
Big Pharma	−0.032	0.076	1					
Weapon	0.152***	0.004	0.121**	1				
Exaggeration	0.164***	0.095	0.056	0.087*	1			
FilmYourHospital	0.134**	0.058	0.090*	−0.022	0.182***	1		
GMO	0.080*	0.043	0.019	0.032	0.099*	0.176***	1	
Vaccines	−0.212***	0.128*	0.593***	0.129***	−0.028	−0.172***	−0.027	1

GMO: genetically modified organism.

* *p* < .05. **p < .01. ***p < .001.

#### Increase Throughout the Pandemic

The second group includes the Big Pharma and the vaccines-related conspiracy theories. The Big Pharma conspiracy fluctuated from March 2020 to July 2021 and then began to rise in frequency (see Figure 1). The frequency of the vaccines-related tweets was constant and very low until October 2020. Then it went up and remained stable until June 2021 at a higher level, after which it began to rise sharply. From Table 3, we see a positive and significant correlation (*r* = .593, *p* < .001) between these conspiracies time series.

#### Persistent Theories

The third group are the exaggeration and the Bill Gates conspiracy theories. The exaggeration conspiracy remained at a high level from March 2020 to January 2021, then declined until June 2021, then sharply reached previous high levels again in August 2021, and declined again (see Figure 1). However, it still remained at a high level in November 2021. The conspiracy on Bill Gates appeared in 2021 and remained at a relatively high level with some fluctuations.

#### Multiple Peaks

The fourth group are the GMO and the biological weapon conspiracies. Both reached a peak at the beginning of the pandemic and sharply declined afterwards to negligible levels (see Figure 1). However, the biological weapon theory peaked again in July 2021 with the peak being higher than in 2020. The GMO theory peaked in August 2021, though the second peak was lower. Afterwards, they both sharply returned to relatively low values.

### Correlation and Cross-Correlation Between the Daily Frequency Conspiracies Tweet Time Series

We conduct a correlation (see Table 3) and a cross-correlation analysis to see how the conspiracy theories are related to each other. While the correlation between weapon conspiracy and vaccines-related conspiracies is significant and positive (*r* = .129, *p* < .001, see Table 3), the cross-correlation between the weapon tweet frequency at time *t* and the vaccines tweet frequency at time *t* *+* *7* is much higher with a coefficient of 0.283. This result suggests that a higher tweet frequency of the weapon conspiracy at time *t* leads to a higher vaccine conspiracy tweet frequency 7 days later (*t* *+* *7*). Bill Gates and weapon have no correlation between them (see Table 3), but the cross-correlation suggests that there is a positive correlation with a coefficient of 0.214 between the weapon at time *t* and the Bill Gates at time *t* *+* *7*. FilmYourHospital and GMO have a significant correlation coefficient of 0.176 (see Table 3), the cross-correlation suggests a higher coefficient of 0.593 between GMO at time *t* and FilmYourHospital 2 days later (*t* *+* *2*), meaning higher GMO tweet frequency leads to higher FilmYourHospital tweet frequency 2 days later. The 5G and Big Pharma have no significant correlation between them (see Table 3), but there is a cross-correlation with a coefficient of −0.247 between 5G at time *t* and Big Pharma at time *t* *+* *5*, and cross-correlation of −0.22 between 5G at time *t* *+* *4* and Big Pharma at time *t*, meaning that a higher tweet frequency of one of the conspiracies leads to a lower frequency in the second a few days later. The 5G and vaccines conspiracies are negatively significantly correlated (*r* = −.212, *p* < .001, see Table 3). The cross-correlation suggests a coefficient of 0.25 at lag + 4 and lag −4, meaning that a higher vaccines tweet frequency at time *t* leads to a lower 5G tweet frequency 4 days later, and vice versa (higher 5G tweet frequency leads to lower vaccines tweet frequency 4 days later).

### Association Between the Frequency of Conspiracy Theory Tweets and the Number of New COVID-19 Cases

We have used Stata for the time series regression. Table 4 summarizes the regression results. The number of COVID-19 cases has a different explanatory power for various conspiracies. The models provide an *R*^2^ between 0.00 and 0.52. The number of COVID-19 cases is found to have a positive and significant effect on the Big Pharma, Bill Gates, vaccines-related, and exaggeration conspiracies. Meaning that 1% increase in the number of cases is associated with 0.27%, 0.77%, 0.33%, and 0.63% increase in the tweet frequency a week later for the Big Pharma, Bill Gates, exaggeration, and vaccines-related conspiracies, respectively. The number of new cases has a negative and significant effect on the 5G, weapon, and the FilmYourHospital conspiracies. Meaning that 1% increase in the number of cases is associated with a decrease of 0.09%, 0.1%, and 0.84% in the tweet frequency a week later for the 5G, weapon, and the FilmYourHospital conspiracies, respectively. The number of new cases does not have a significant effect on the GMO conspiracy.

**Table 4.** OLS Regression Results.

	(1)	(2)	(3)	(4)	(5)	(6)	(7)	(8)
	Ln 5G_t_	Ln BigPharma_t_	Ln BillGates_t_	Ln Weapon_t_	Ln Exaggeration_t_	Ln FilmYourHospital_t_	Ln GMO_t_	Ln Vaccines_t_
Ln(casest_*-7*_)	−0.09** (−2.60)	0.27*** (13.15)	0.77*** (9.19)	−0.10*** (−5.27)	0.33*** (11.58)	−0.84*** (−9.21)	−0.01 (−0.49)	0.63*** (23.56)
_cons	6.68*** (15.20)	1.93*** (7.33)	−4.05*** (−3.68)	6.70*** (27.92)	1.91*** (5.26)	12.41*** (10.53)	3.08*** (15.02)	−3.91*** (−11.58)
*N*	671	671	273	671	671	598	671	671
*R* ^2^	.02	.29	.23	.05	.41	.22	.00	.52

OLS: ordinary least squares; GMO: genetically modified organism.

The number of new COVID-19 cases a week before as an explanatory variable for the tweet frequency of each conspiracy theory. *t* statistics in parentheses.

* *p* < .05. **p < .01. ***p < .001.

## Discussion

Our findings allow us to identify certain patterns and draw several conclusions on the development of the conspiracy theories.

### Peak at the Beginning of the Pandemic

Our first finding is that some conspiracies were actual only at the beginning of the pandemic and sharply declined afterward. One of them was the 5G conspiracy—the conspiracy theory mainly connected with the risk perceptions of digitalization in general. This was the topic which raised a lot of attention under conditions of uncertainty. Then it became not actual. Another conspiracy which was actual only at the beginning of the pandemic was the FilmYourHospital conspiracy theory. We think this has a logical explanation, that hospitals eventually began to fill up and there were no more empty beds to film. We suppose that both conspiracies related to the dread risk bias, namely that the COVID-19 pandemic was new, and its reasons were unclear. As we see from Table 4, the frequency of the discussion of both conspiracies declined with the number of new COVID-19 cases.

### Increase Throughout the Pandemic

Our second finding is that the frequency of the discussion of some theories increased with the evolvement of the pandemic. Moreover, they began to play a major role only recently with the beginning of active and mass vaccination. These include conspiracy theories about vaccines and the Big Pharma. However, they were gaining by far not as much attention as 5G at the beginning of the pandemic. As we see from Table 4, the frequency of the discussion of these conspiracies increased with the number of new COVID-19 cases. These findings are consistent with the threat and distrust heuristic, which refers to industrial power and related concerns about the role of the big industry, as well as the argument of pure progress, which causes different doubts among the population.

### Persistent Theories

Our third finding is that some theories remained stable with some fluctuations over time. The theory of Bill Gates' role began to gain momentum in 2021 and remained at a consistently high level. This may be due to the introduction of digital certificates and the use of Bill Gates by conspiracy theorists as the embodiment of digital slavery. This discourse is again related to the threat and distrust heuristic as well as the use of the pure progress argument. The discussion of the exaggeration conspiracy theory remained persistent throughout the pandemic. This may indicate people’s distrust of statistics. From Table 4, we see that both conspiracies were discussed more with the increase in the number of new cases.

### Multiple Peaks

Our fourth finding is that some theories had double peaks. The first peak was at the beginning of the pandemic, the second peak was at the beginning of the active and massive introduction of vaccines. Such theories include conspiracy theories about GMO and biological weapon. We assume that people first tried to explain the origin of the virus with these theories and then began to call the vaccine GMO and biological weapon. We also see that these theories faded as quickly as they emerged after they peaked. After reaching the peaks, the theories only played a negligible role. From Table 4, we see that the discussion of the GMO conspiracy was not driven by the number of new COVID-19 cases. The discussion of the weapon conspiracy was negatively affected by the number of new cases, but with a low *R*^2^. These were rather the events—the appearance of the pandemic as a new, unexpected event and the introduction of a vaccine, as outlined above, that had an effect on the emergence and development of these conspiracies.

### Policy Recommendations

The persistence of the COVID-19 conspiracy theories could be one of the reasons of low COVID-19 vaccine acceptance and high vaccine hesitancy around the globe (Hassan et al., 2021; Rosiello et al., 2021). This makes the findings important for policy makers, who can identify theories that remain persistent despite 2 years of pandemic development and anti-conspiracy news campaigns, and to develop measures aimed specifically at combating the remaining conspiracies.

## Conclusion

In this article, we have analyzed 1.269 million extracted tweets using keywords related to the most common COVID-19 conspiracy theories. The results of the analysis have helped us identify patterns and categorize existing conspiracy theories into four groups. The first—5G and FilmYourHospital—played a major role at the beginning of the pandemic and then declined sharply. The second—vaccines and Big Pharma—began to play a major role later as vaccines began to be actively introduced. The third—exaggeration and the role of Bill Gates—remained relatively high over a long period of time with some fluctuations. The fourth—GMO and biological weapon—had two peaks and were driven by two events—the emergence of the pandemic and the active start of the vaccination campaign. We also find that the number of cases was a significant predictor to the conspiracy tweet frequency a week later for seven out of eight conspiracies.

This shows that many people react to new, unexpected, and incomprehensible risks by resorting to conspiracy narratives. People refer to the heuristic-systematic model in response to the threat and rely on a set of heuristics such as the threat and distrust heuristic. However, when the picture becomes clearer and more reliable and clear information emerges, and many are confronted with the virus themselves, most narratives fade away on their own. But there are some that remain quite persistent.

The main limitation of the study is that we do not distinguish between the types of tweets we have collected. We could have collected tweets supporting the conspiracy, opposing the conspiracy or irrelevant to the conspiracy. However, based on our collection terms and method of collection, we assume for this study that most of the collected tweets are related to the discussion on our conspiracy theories of interest. Exploring the sentiment of the analyzed tweets and categorizing them into supporters, opponents, and neutrals in relation to the conspiracy theories could be a topic for future research.
